# Supplementary material for: The Autophagy Inhibitor Chloroquine, Alone or in Combination with mTOR Inhibitors, Displays Anti-Tumor Effects in In Vitro and In Vivo Lung Carcinoid Models
Source: Cancers (Basel). 2021 Dec 16;13(24):6327. doi: 10.3390/cancers13246327 (PMC8699234; doi:10.3390/cancers13246327)

cancers

MDPI

Article

The Autophagy Inhibitor Chloroquine, Alone or in Combination with mTOR Inhibitors, Displays Anti-Tumor Effects in In Vitro and In Vivo Lung Carcinoid Models

Adi Knigin, Shani Avniel-Polak, Gil Leibowitz, Kira Oleinikov, David J. Gross and Simona Grozinsky-Glasberg

Supplemental materials

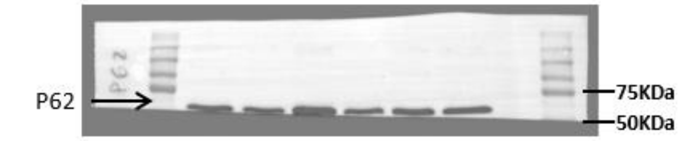

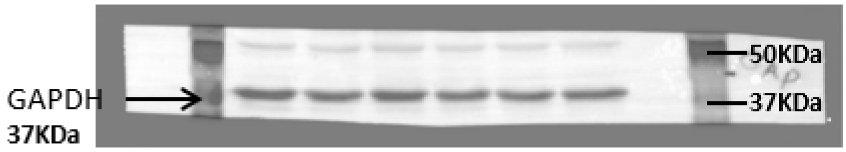

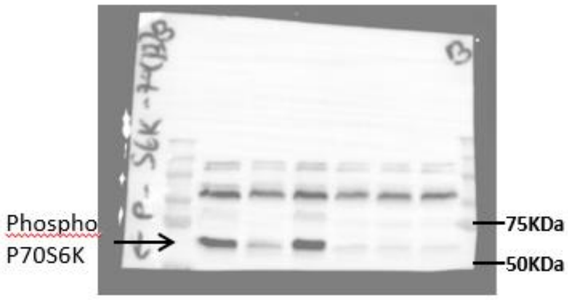

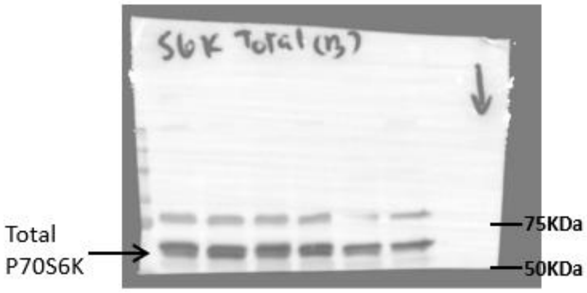

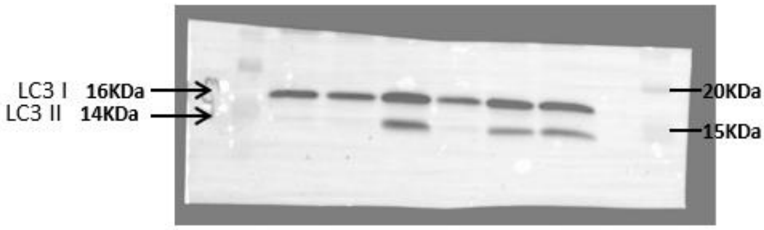

Supplement: Supplementary file 1 [file cancers-13-06327-s001.zip › cancers-1429126 supplemental materials .pdf]
